# Primary Testicular Pre-B Lymphoblastic Lymphoma

**Published:** 2016-04-24

**Authors:** Fariba Binesh, Mohammad Forat Yazdi, Alireza Jenabzadeh, Somayeh Hosseini, Roghayeh Massumi

**Affiliations:** 1 Department of Pathology, Shahid Sadoughi University of Medical Sciences, Yazd, Iran; 2 Department of Hematology and Oncology, Shahid Sadoughi University of Medical Sciences, Yazd, Iran; 3 Department of Pediatric Oncology, Shahid Sadoughi University of Medical Sciences, Yazd, Iran; 4 Hematology and Oncology Research Center, Shahid Sadoughi University of Medical Sciences, Yazd, Iran

**Keywords:** Lymphoblastic lymphoma, Testis, Children

## Abstract

Primary testicular lymphoblastic lymphoma is a rare entity. We report a case of a 13-year-old boy referred with unilateral testicular swelling. After preliminary work-up orchiectomy was performed Histopathology detected primary testicular lymphoblastic lymphoma. Lymphoblastic lymphoma should be considered in the differential diagnosis of testicular masses in children.

## INTRODUCTION

Lymphoblastic lymphoma is usually reported in children and adolescents. In approximately half of the cases mediastinal mass is present. Malignant lymphoma constitutes 5% of all testicular malignancies.[1] In adults primary diffuse large B cell lymphoma is the most frequent lymphoma, whilst the majority of testicular lymphomas in children express secondary involvement by Burkitt lymphoma, diffuse large B cell lymphoma, or lymphoblastic lymphoma.[2] Testicular infiltration occurs more commonly during disease dissemination or relapse. Primary testicular lymphoblastic B-cell lymphoma is a rare entity. Here we report a case of primary testicular precursor B-lymphoblastic lymphoma in a teen ager boy.

## CASE REPORT

An otherwise healthy 13-year-old boy presented to a local private clinic for a sudden unilateral enlargement of the left testis. His past medical history was unremarkable. There was no history of trauma or fever. Examination of the scrotum showed moderate swelling and tenderness of the left testicle; right testis was normal. There was no lymphadenopathy. Ultrasound revealed a highly vascularized, hypoechoic lesion, completely infiltrating the testis. The clinical impression was testicular tumor and a total orchiectomy was done through inguinal approach. On gross examination, a complete replacement of the testis by a whitish, relatively homogenous tumor was observed. Microscopically, there was a diffuse pattern of proliferation of relatively small lymphoid cells, between and displacing seminiferous tubules (Fig. 1). The neoplastic cells had scanty cytoplasm and nucleus with delicate nuclear membrane convolutions. The chromatin was finely stippled, and nucleoli were inconspicuous. Mitotic activity was extremely high. The neoplastic cells expressed CD 45, TdT, CD10, CD99, HLA DR, and CD34; and were negative for CD117, CD2, CD 20 and CD3. Ki67 was positive in 90% of tumor cells (Fig. 2). He had no superficial lymphadenopathy or hepatosplenomegaly. Chest CT scans and abdominal ultrasound did not indicate mediastinal and retroperitoneal enlarged lymph node. Laboratory test results were within normal range. The result from cerebrospinal fluid and bone marrow aspiration showed that there was no infiltration. He was diagnosed with B lymphoblastic lymphoma, Ann Arbor stage I.

**Figure:**
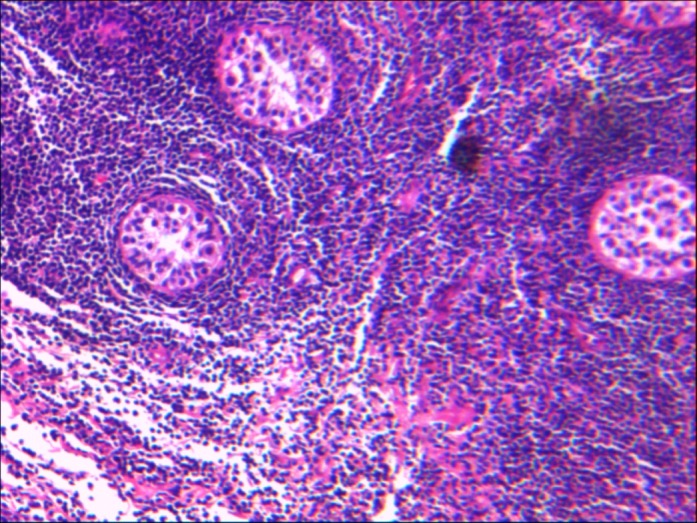
Figure 1:Shows a diffuse and monomorphic tumor cells, between and displacing seminiferous tubules (H and E X10).

**Figure:**
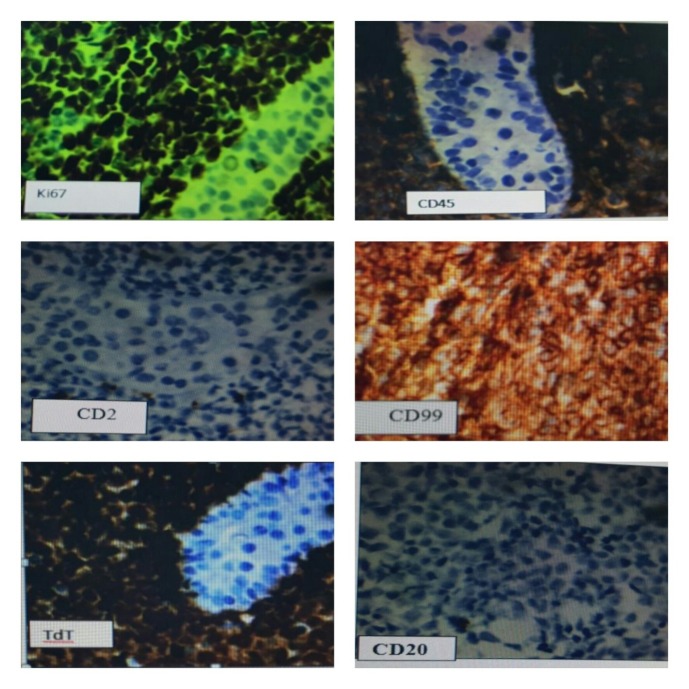
Figure 2:Neoplastic cells expressed CD 45, TdT, CD99 and were negative for CD2 and CD 20 . Ki67 was positive in 90% of tumor cells (IHC stain).

A multidisciplinary consultation with urologists, oncologists, radiation oncologists and hematologists was done. Despite the absence of bone marrow infiltration and other mass lesions, the oncologist chose to treat the patient with a high dose combined systemic and intra-thecal chemotherapy, followed by intensive consolidation therapy (chemotherapy, Protocol M). His parents refused radiation therapy. At the completion of the consolidation phase of treatment a PET scan was done which showed no abnormality. After 8-month of follow-up, the patient is without disease.

## DISCUSSION

Primary testicular lymphoblastic lymphoma is labelled if the disease is restricted to the testis and no signs of bone marrow involvement are present at diagnosis or develop during follow-up. In our case no lymph node, central nervous system, or bone marrow involvement was detected at diagnosis. There are few reports in literature about primary testicular B-lymphoblastic lymphoma.[3-5] Literature review disclosed five cases of primary testicular pre B lymphoblastic lymphoma.[3-6] Primary testicular lymphoma usually presents as a unilateral testicular mass with occasional bilateral involvement. In 10-18% of cases there is synchronous or asynchronous involvement of contra lateral testis. [7] Scrotal ultrasound can be used for initial evaluation and follow-up after therapy to determine recurrence. Sonography usually reveals a focal hypoechoic mass without a distinctive capsule or diffuse enlargement and decreased echogenicity of the whole of the testis which is different from the normal testis.[8] However, primary testicular lymphoma is not associated with any specific findings at ultrasound and is not different from other testicular tumors.

A round blue cell tumor of testis has following major differential diagnoses as lymphoblastic lymphoma, Burkitt lymphoma, blastic variant of mantle cell lymphoma, small lymphocytic lymphoma, myeloid sarcoma, Ewing sarcoma/peripheral neuro-ectodermal tumor, neuroblastoma, and embryonal rhabdomyosarcoma.[9] The age of the patient, clinical presentation, morphologic evaluation and, finally immunohistochemistry allow discrimination among these entities. Similarly in our case, immunohistochemistry was helpful in achieving the correct diagnosis. Once diagnosis of testicular lymphoblastic lymphoma has been made, a complete staging is essential. Contrary to the localized presentation of disease, primary testicular lymphoblastic lymphoma is a systemic process and treatment should be provided to minimize recurrence. Result of one study showed that chemotherapy without radiation therapy for localized lymphoblastic lymphoma in children led to a relapse rate of 30%.[10] In the current case, the oncologist chose to treat the patient with a high dose combined systemic and intra-thecal chemotherapy, followed by intensive consolidation therapy. However, his parents refused radiation therapy. Our therapeutic approach was similar to Garcia et al.[6] In conclusion, though very rare, primary testicular lymphoblastic lymphoma should be considered in the differential diagnoses of testicular masses, especially in young patients.

## Footnotes

**Source of Support:** Nil

**Conflict of Interest:** None declared

